# A Remarkable Case of Opium Poisoning

**Published:** 1879-09

**Authors:** Gustav Keitz

**Affiliations:** 666 North Rampart Street; New Orleans, La.


### ARTICLE IX.

### *A Remarkable Case of Opium Poisoning.*

The following case offers several points of interest:

Mrs. M. E. K., æt. 27 years, married five and a half years; of small size, but well nourished.

*Antecedents.* — She has had two children at full term; miscarried twice. Her first pregnancy was a good one; she enjoyed good health throughout, and gave birth to a healthy girl, which died last year of yellow fever at an age of 3 years and 2 months. The second pregnancy was not so easy; she had convulsions (puerperal) towards the end of the sixth month, from which she recovered, and gave birth to a girl at full term. During her entire second pregnancy she was in ill-health, had little appetite and was very anæmic. The two miscarriages took place, one before she became pregnant with her second child (which is now living, and a fine, healthy girl), and the other thereafter.

At present she is in the ninth month of another pregnancy. Since the fourth month of her present pregnancy she has suffered with false pains, which recurred regularly towards the beginning of each subsequent month, but which, under good care and judicious sedative treatment, left her after an illness of about two days each time. She had at times slight suppression of urine, with traces of albumen.

These attacks seemed to increase in violence each time, and on the first of June, 5 p. m., she had a puerperal convulsion. I

put her at once under chloroform, and administered a solution of hydrate of chloral, 2.0, Batley's sedative gtts. xv, in water, 15.0, per rectum, which dose was repeated two hours later. At the same time I applied powerful rubefacients along the spine, and especially over the region of the kidneys. She commenced to pass urine freely and copiously, and no convulsion has as yet returned.

Her last attack occurred on July 3d at 9 p. m. I applied rubefacients to the spine as before, and gave, by rectum, a dose of chloral and Batley's sedative as mentioned above, which was repeated at 12 o'clock the same night and at 7 and 8:30 the next morning.

She slept a little during the night, and in the morning from 8:30 till 12:45 p. m. (four hours and fifteen minutes). The last sleep was very refreshing. She drank a little iced wine, but ate nothing.

In the evening, at 6 o'clock, she partook an ordinary wine-glassful of ice-cream. Half an hour later she was taken with retching, and ejected about two teaspoonfuls of what appeared to be curdled milk. The pains, which had left her completely that morning, commenced again, steadily increasing in violence.

At 7 p. m. I administered morph. sulph. 0.01, subcutaneously in the *left* arm, which, not having given the slightest relief after twenty minutes, was followed by hydrate chloral 1.25, Batley's sedative 0.6, and water 10.0, per rectum. No effect was noticed until twenty minutes after the last dose (*i.e.*, forty minutes after the administration of the morphine in the left arm), when all at once the lady's face became hot and flushed, the breathing very slow (but not stertorous), the pulse quick and small and *both* pupils contracted to the size of a pin's head. Respiration, 10 ; pulse, 120. Rate of pulse to respiration : 12 : 1.

I became at once aware of the fact that I had a case of opium (and chloral) poisoning on hand.

I rubbed the patient's face, neck and spine with ice, and prescribed an ointment composed of extr. belladonnæ 3.75, cerati camphoræ 15.0. I did not want to use atropinum hypodermically, fearing that I might run into the opposite extreme ; but wished to secure a very slow and gradual absorption of the belladonna.

Of this ointment I applied about 4.0 to the back, on *both sides* of the spine, every twenty minutes, closely watching its effect on the pupils.

To my great astonishment and surprise, ten minutes after the second application, the *right* pupil dilated rapidly, and was expanded to its utmost in a remarkably short time (eight minutes later), while the *left pupil remained contracted as it was before the application of the belladonna, viz., to the size of a pin's head.* I desisted from the further application of the belladonna; the ice was continued.

Three hours and twenty minutes after the commencement of the opium narcosis (11 p. m.) the lady was so far resuscitated as to be able to answer questions rationally, she complained of headache, drowsiness and dimness of sight. The pupils remained as described, viz., the right dilated to its utmost degree, the left contracted to the size of a pin's head, until half-past three in the morning, so that by 4:30 they were of normal size, and responded to the light again.

The fœtal movements were not felt until about 11 o'clock a. m., July 5th, and at night they became as strong as ever they were before.

The noteworthy points in this case are:

1. A comparatively small dose had produced poisoning. The interval between the last dose in the morning and the first dose at night was 11 hours.

2. Why did the right pupil show the effect of the belladonna, while the left one remained contracted, and this without the slightest alteration for about 6½ hours? The morphine solution (10 minims of a solution containing morph. sulph. 0.06 in 60 minims of water) was injected into the left arm, the belladonna ointment was applied equally over and to both sides of the spine. What was the cause of the persistent difference between the two pupils, since the drugs act constitutionally?

3. Not the slightest harm has so far resulted to the fœtus.

GUSTAV KEITZ, M.D.,

666 North Rampart Street.

NEW ORLEANS, LA.,

July 9th, 1879.

